# Association between Malnutrition and Migraine Risk Assessed Using Objective Nutritional Indices

**DOI:** 10.3390/nu15173828

**Published:** 2023-09-01

**Authors:** Jong-Ho Kim, Young-Suk Kwon, Jae Jun Lee, Sang-Hwa Lee, Jong-Hee Sohn

**Affiliations:** 1 Department of Anesthesiology and Pain Medicine, Chuncheon Sacred Heart Hospital, Hallym University College of Medicine, Chuncheon-si 24253, Republic of Korea; poik99@hallym.or.kr (J.-H.K.); gettys@hallym.or.kr (Y.-S.K.); iloveu59@hallym.or.kr (J.J.L.); 2 Institute of New Frontier Research Team, College of Medicine, Hallym University, Chuncheon-si 24252, Republic of Korea; neurolsh@hallym.or.kr; 3 Department of Neurology, Chuncheon Sacred Heart Hospital, Hallym University College of Medicine, Chuncheon-si 24253, Republic of Korea

**Keywords:** migraine, nutrition, the controlling nutritional status score, the prognostic nutritional index score

## Abstract

Dietary triggers are frequently linked to migraines. Although some evidence suggests that dietary interventions might offer a new avenue for migraine treatment, the connection between migraine and nutrition remains unclear. In this study, we explored the association between nutritional status and migraines. Clinical data spanning 11 years were sourced from the Smart Clinical Data Warehouse. The nutritional statuses of 6603 migraine patients and 90,509 controls were evaluated using the Controlling Nutrition Status (CONUT) score and the Prognostic Nutrition Index (PNI). The results showed that individuals with mild, moderate, and severe malnutrition were at a substantially higher risk of migraines than those with optimal nutrition, as determined by the CONUT score (adjusted odds ratio [aOR]: 1.72, 95% confidence interval [CI]: 1.63–1.82; aOR: 5.09, 95% CI: 4.44–5.84; aOR: 3.24, 95% CI: 2.29–4.59, *p* < 0.001). Similarly, moderate (PNI: 35–38) and severe (PNI < 35) malnutrition were associated with heightened migraine prevalence (aOR: 4.80, 95% CI: 3.85–5.99; aOR: 3.92, 95% CI: 3.14–4.89, *p* < 0.001) compared to those with a healthy nutritional status. These findings indicate that both the CONUT and PNI may be used as predictors of migraine risk and underscore the potential of nutrition-oriented approaches in migraine treatment.

## 1. Introduction

Migraine is a prevalent primary headache disorder that is frequently linked to dietary triggers. Although numerous studies have identified a connection between dietary triggers and migraines, there is a pressing need for verification based on high-quality longitudinal studies [1,2,3,4].

Certain foods and dietary supplements, including magnesium, coenzyme Q10, feverfew, riboflavin, phycocyanin, and vitamin D, are purported to prevent or alleviate migraines. These alternatives may be particularly beneficial to patients who respond poorly to conventional drug therapy, such as adolescents, pregnant or lactating women, and individuals with contraindications to standard pharmacological treatments. Although these options have favorable safety profiles, evidence suggests that they have limited efficacy [5]. Moreover, despite the reported benefits of several dietary interventions in migraine prevention and treatment, robust evidence to strongly advocate dietary interventions as an approach for migraine management remains sparse [5,6,7,8,9,10,11,12,13,14,15].

The role of diet in migraine is multifaceted, potentially influencing mechanisms such as the modulation of neuropeptides, neuroreceptors, ion channels, the sympathetic nervous system, and cerebral glucose metabolism, and possibly instigating inflammation, nitric oxide release, and vasodilation [16]. Notably, the calcitonin gene-related peptide (CGRP), a pivotal neuropeptide for pain transmission that is released from trigeminal termini during migraine episodes [17], appears responsive to dietary nutrients. The influence of CGRP on the gut–brain–microflora axis is essential, and dietary elements disrupting calcium signaling can impact its secretion while enhancing mitogen-activated kinase phosphatases [18].

However, despite preliminary evidence suggesting the potential benefits of avoiding dietary triggers, modulating nutrient consumption, and following specific diets to alleviate migraine symptoms, the intricate relationship between migraine and nutrition has yet to be fully grasped. In clinical scenarios, it is often difficult to objectively evaluate nutritional health using basic tools such as dietary diaries or weight checks. Objective nutritional indices such as the Controlling Nutritional Status (CONUT) score and the Prognostic Nutritional Index (PNI) have been established as effective tools in this context. These indices utilize parameters that offer streamlined assessment methods based on indicators such as serum albumin, cholesterol, and peripheral blood lymphocyte levels [19,20].

In this study, we investigated the potential correlation between migraines and nutritional health using an 11-year dataset obtained from the Smart Clinical Data Warehouse (CDW), employing CONUT and PNI as tools to elucidate the association between nutritional status and migraines.

## 2. Materials and Methods

### 2.1. Subjects

We analyzed clinical data extracted from the Smart CDW at Hallym University Medical Center, covering the period from January 2013 to March 2023. The Smart CDW, supported by Qlik-View Elite Solution (Qlik, Lund, Sweden), is employed across all five Hallym University Medical Center hospitals. This system enables the integration and examination of text-based data in tandem with structured data from electronic medical records.

The eligibility criteria for migraine patients included age between 20 and 80 years, a diagnosis of migraine confirmed by board-certified neurologists, a reference to the International Classification of Diseases, 10th revision (ICD-10) code G43 in their medical records, and a history of at least two outpatient visits or a single admission to the neurology department. The control group consisted of individuals aged 20 to 80 years who underwent general health screenings at a health promotion center. Those with a prior history of headaches or migraines were identified through a basic pre-checkup questionnaire. Individuals visiting our medical center explicitly for headache or migraine treatment were excluded. The Clinical Research Ethics Committee of Chuncheon Sacred Heart Hospital, Hallym University, granted approval for the study protocol (IRB no. 2023-07-013). Given that the study employed only de-identified data, the review board waived the need for informed consent.

### 2.2. Clinical Data Collection

From the CDW, we gathered demographic details such as age and gender; weight and height measurements to compute the body mass index (BMI); information on comorbidities including diabetes mellitus, hypertension, dyslipidemia, angina, atrial fibrillation, heart disease, cerebrovascular diseases, chronic pulmonary disorders, renal failure, chronic hepatitis, anxiety disorder, depression, sleep disorders, and menopause; and laboratory data collected within one month both preceding and following the migraine diagnosis, as well as during the health check-up. For individuals with multiple laboratory test outcomes within the month-long window around their migraine diagnosis, the mean value was utilized.

Comorbidities were characterized using pertinent ICD-10 codes in the database, including diabetes mellitus (E10–14), hypertension (I10–15), dyslipidemia (E78), angina (I20, I24, and I251), atrial fibrillation (I480–482 and I489), heart disease (I05–09, I21–23, I30–47, and I49–52), cerebrovascular diseases (G45–46 and I60–69), chronic pulmonary disorders (J40–47), renal failure (N03 and N18–17), chronic hepatitis (B18, I85, and K70–74), anxiety disorder (F41), depression (F31–34, F412, and F432), sleep disorders (F51, G258, and G47), and menopause (M800, M010, N924, and N95).

### 2.3. Nutritional Status Assessments

The nutritional health of migraine patients and control participants was assessed using the CONUT score and the PNI. The CONUT score is determined based on parameters including serum albumin concentration, total peripheral lymphocyte count, and serum total cholesterol concentration. Each of these metrics was gauged against its respective normal range, culminating in a comprehensive score. Depending on this score, patients were segmented into normal (score 0–1), mild (2–4), moderate (5–8), and severe (9–12) malnutrition risk categories [21]. The PNI was calculated as 5 × Lymphocyte count (109/L) + 10 × Serum albumin concentration (g/dL), where PNI > 38 indicated normal nutritional health, and 35 < PNI < 38 and PNI < 35 indicated moderate and severe malnutrition risk, respectively. There was no “mild” malnutrition risk category [22]. These nutritional assessment techniques are described in greater detail in Table A1 [23,24].

### 2.4. Statistical Analyses

Continuous variables are presented as medians and interquartile ranges (IQRs), and categorical variables are expressed as counts and percentages. The Mann–Whitney test was used to compare continuous data between the migraine and control groups. For categorical data comparisons, the chi-squared test was employed. Both the CONUT and PNI scores were analyzed in two ways: as continuous variables and grouped either into four categories (absent, mild, moderate, or severe) or three categories (absent, moderate, or severe).

Multivariate analyses were performed to explore the connection between malnutrition, in terms of the nutritional indices (CONUT and PNI), and migraine. Odds ratios (ORs) and their 95% confidence intervals (CIs) were calculated using logistic regression. These ORs indicate the risk of malnutrition in relation to exposure compared to the risk without exposure. Fully adjusted ORs for both CONUT and PNI were determined for each factor, including migraine. The ORs were adjusted for all variables, as well as for those selected through backward elimination.

All *p* values were two-tailed. Bonferroni correction was implemented to manage multiple testing, with statistical significance evaluated at *p* < 0.025, which was derived by dividing the usual *p* threshold (0.05) by the number of tests (CONUT and PNI). SPSS v24.0 software (IBM Corp., Armonk, NY, USA) was used for statistical analyses.

## 3. Results

### 3.1. Subject Characteristics

**Figure 1:**
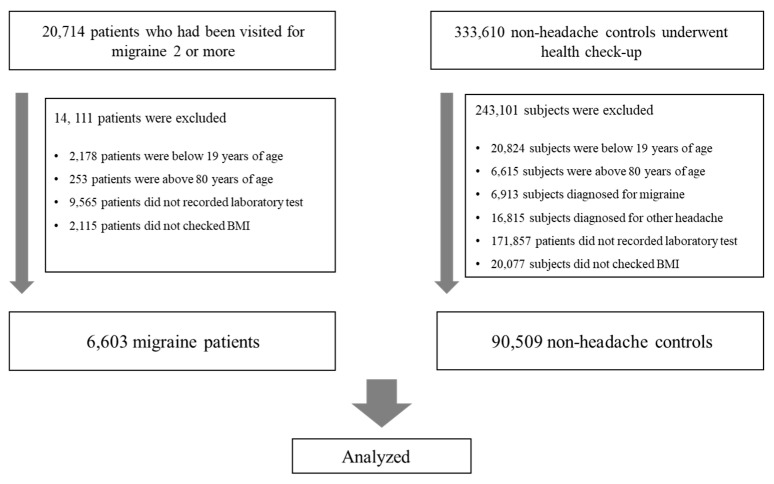
Flow chart of the enrollment process. BMI, body mass index.

From January 2013 to March 2023, 20,714 patients with a migraine diagnosis were included in the study, and 333,610 control subjects were identified from the same database. The participant inclusion flow is depicted in Figure 1. Upon applying the exclusion criteria, the final cohort consisted of 6603 migraine patients and 90,509 non-headache control individuals. Excluded from the migraine group were 2178 patients under 20 years, 253 over 80 years, 9565 with incomplete laboratory data, and 2115 without recorded BMI. Excluded from the control group were 20,824 subjects under 20 years, 6615 over 80 years, 171,875 with missing laboratory results, 23,728 with a history of headaches or migraines based on preliminary questionnaires or those seeking headache or migraine treatment, and 20,077 without a BMI record. Comprehensive clinical details of the participants are tabulated in Table 1.

### 3.2. Comparison of Nutritional Status between Patient Groups

Among the 6603 patients analyzed, 3750 patients (56.8%) had CONUT scores from 0 to 1, indicating normal nutritional status; 2455 patients (37.2%) had scores from 2 to 4, indicating mild malnutrition; 355 patients (5.4%) patients had scores from 5 to 8, indicating moderate malnutrition; and 43 patients (0.7%) had scores from 9 to 12, indicating severe malnutrition. For PNI scores, 6356 patients (96.3%) had a value ≥38, reflecting normal nutritional status. Only 128 (1.9%) had scores between 35 and 38 (moderate malnutrition), and 119 (1.8%) had scores below 35 (severe malnutrition).

Comparing the nutritional metrics between migraine and control participants, significant differences emerged for both CONUT [1 (CI: 0–2) for both groups, *p* < 0.001] and PNI [52.1 (CI: 47.2–56.3) vs. 54.7 (CI: 51.5–57.8), *p* < 0.001). When comparing categorized groups, the migraine cohort displayed higher prevalence rates in the moderate or severe categories based on both CONUT and PNI scores. The malnutrition proportion in migraine patients was 43.3% as determined by the CONUT system and 3.7% by the PNI system. Given the modest number of malnutrition cases (3.7%) according to the PNI system, scores were further divided into tertiles, which revealed a higher proportion of migraine patients in the lowest PNI tertile compared to controls (Table 2).

### 3.3. Association between Nutritional Status and Migraine

In the multivariable analysis, individuals with mild, moderate, or severe malnutrition, as indicated by the CONUT score, had a significantly elevated risk of migraine (aOR for mild: 1.70, 95% CI: 1.61–1.80, *p* < 0.001; for moderate: 4.99, 95% CI: 4.36–5.72, *p* < 0.001; for severe: 3.18, 95% CI: 2.25–4.51, *p* < 0.001), in comparison to those with normal nutritional status. We also noted that moderate (35 < PNI < 38) and severe malnutrition (PNI < 35), as classified by the original scoring system, were associated with a higher migraine prevalence (aOR for moderate: 4.76, 95% CI: 3.82–5.94, *p* < 0.001; for severe: 3.88, 95% CI: 3.11–4.84, *p* < 0.001) compared with normal nutritional status (PNI > 38), after accounting for potential confounders. When PNI was categorized into tertiles, individuals in the highest tertile (PNI > 56.59) exhibited a decreased migraine risk relative to those in the lowest tertile (PNI < 52.55) (aOR for highest tertile: 0.43, 95% CI: 0.40–0.46, *p* < 0.001; for second tertile: 0.49, 95% CI: 0.46–0.53, *p* < 0.001) in the multivariable analysis (Table 3).

**Figure 2:**
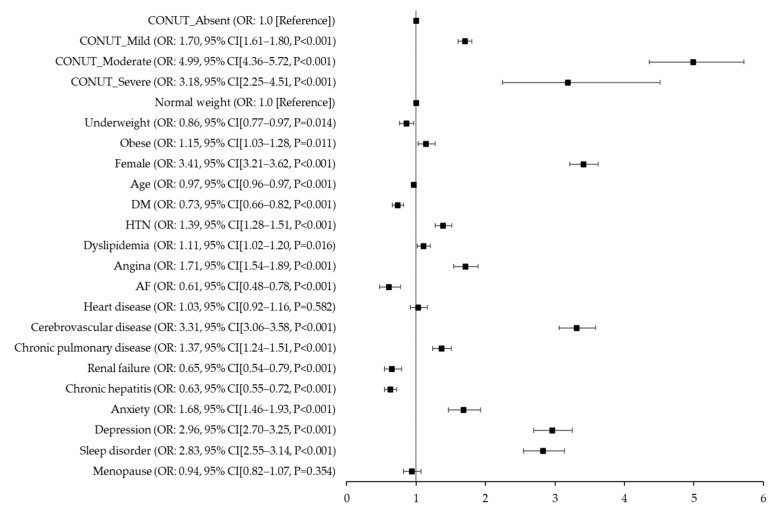
Fully adjusted odds ratios for migraine occurrence based on multivariate analysis with clinical variables and Controlling Nutrition Status (CONUT) scores. CONUT, Controlling Nutritional Status; DM, diabetes mellitus; HTN, hypertension; AF, atrial fibrillation.

**Figure 3:**
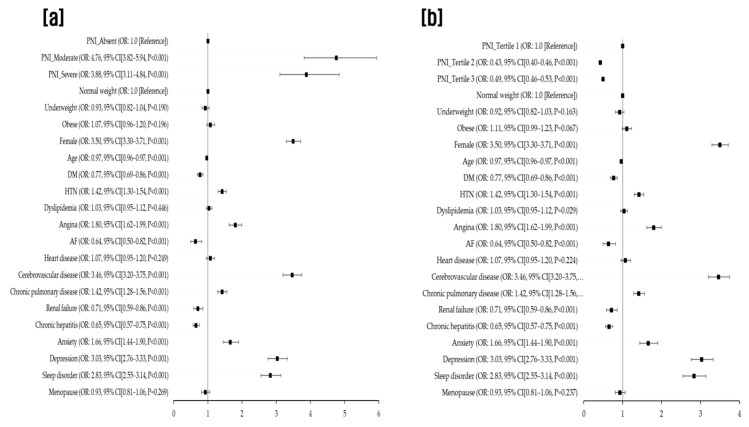
Fully adjusted odds ratios for migraine occurrence based on multivariate analysis incorporating clinical variables and Prognostic Nutrition Index (PNI) scores evaluated using both the original scoring system (**a**) and tertiles (**b**). PNI, prognostic nutrition index; DM, diabetes mellitus; HTN, hypertension; AF, atrial fibrillation.

Figure 2 provides the fully adjusted odds ratios (aORs) for migraine occurrence, as derived from a multivariate analysis incorporating all clinical factors and CONUT scores (Figure 2). Correspondingly, Figure 3 delineates the aORs for migraine onset based on a multivariate analysis that integrated all clinical parameters and PNI scores. In this study, PNI scores are presented categorically, considering both the traditional scoring method and the tertile divisions (Figure 3).

## 4. Discussion

In this study, we explored the relationship between nutritional status and the risk of migraine by employing objective nutritional indices that can be easily measured in clinical practice. An assessment of these indices between migraine patients and control subjects unveiled the more pronounced prevalence of mild to severe malnutrition, as defined by both CONUT and PNI scores, in the migraine cohort. Our multivariable analysis showed that individuals exhibiting mild, moderate, or severe malnutrition, as gauged by the CONUT score, along with those with moderate or severe malnutrition according to the PNI, faced a markedly elevated risk of developing migraines compared with individuals maintaining a normal nutritional status, after accounting for confounding factors. The implications of our results are twofold: malnutrition, as determined through CONUT and PNI scores, appears to be linked with migraine, and both of these scores may hold predictive value for migraine susceptibility.

The CONUT and PNI scores were designed as gauges of the patient’s immune–nutritional condition [19]. Historically, these tools have been utilized to evaluate the nutritional well-being of patients admitted to hospitals and have been useful prognostic tools for a range of ailments, from cardiovascular disease and heart failure to various malignancies [25,26,27,28,29,30,31]. Recent scientific literature has pointed out potential links between these indices and intracranial venous sinus thrombosis. These markers have offered invaluable insights into predicting patient outcomes post-acute ischemic thrombolysis, gauging recovery trajectories after acute ischemic strokes, and discerning the risk of cognitive lapses in patients diagnosed with neurological disorders [32,33,34,35]. However, fewer studies have explored the link between nutritional status and migraines, with only a handful applying objective markers such as CONUT and PNI. One such study assessed nutritional health using PNI to examine variations in daily nutrient intake between migraine sufferers and their non-affected counterparts. This study utilized data sourced from a public US database spanning 1999–2004 and concluded that mild (PNI: 45–50) to moderate or severe malnutrition (PNI < 45) was correlated with a heightened prevalence of severe headaches or migraines (OR: 1.06 or 1.07, respectively) [36]. Despite differences in PNI scoring methodologies between this cited study and our own, the underlying correlations between PNI values and migraine manifestation were consistent with our findings. Notably, in our investigation, PNI categorizations rooted in the foundational literature [22] manifested stark differences in malnutrition ratios among migraine patients when contrasted with CONUT scores. This divergence prompted us to perform an analysis based on tertile classifications. Earlier research employed varied PNI categorizations, ranging from binary divisions derived from receiver operating characteristic curve evaluations to fixed threshold values and tertile groupings [19,34,37,38].

Malnutrition refers to a subacute or chronic state of nutrition characterized by varying degrees of undernutrition and inflammatory activity resulting from inadequate intake or assimilation of nutrients. This state leads to altered body composition, diminished body cell mass, and decreased physical and mental functions [39,40]. Malnutrition is common in various diseases, particularly chronic ones. Its presence can negatively impact the diagnosis, prognosis, and clinical trajectory of both acute and chronic diseases [22,41]. Migraine, a chronic neurological condition with multifactorial etiology, arises from both genetic and environmental influences. Numerous internal and external factors can precipitate migraine triggers, with diet-related triggers being particularly prevalent [42]. Although the precise pathogenesis of migraines remains elusive, the role of nutrition in its onset is pivotal. For example, several vitamins and supplements are recommended for migraine prevention, and certain diets have been reported to mitigate migraine frequency [5,43]. However, data from high-quality randomized controlled trials regarding diet-related triggers is scant. Previous studies have often had small sample sizes and yielded inconclusive results, with a predominant focus on single nutrients or specific diets. Moreover, migraineurs commonly avoid foods perceived as headache triggers, potentially compromising their nutritional balance [44,45]. In this context, there is a pressing need for more in-depth research on the nexus between nutritional status and migraines.

Nutrition stands out as a significant factor in migraine pathogenesis. Past research using 31P-nuclear magnetic resonance imaging has revealed suboptimal brain energy production in areas pertinent to migraine onset [46]. Given that glucose metabolism is central to brain energy synthesis, mitochondrial function and oxidative processes may be instrumental in migraine development. Consequently, specific nutrients beyond carbohydrates can enhance brain energy balance by bolstering mitochondrial function and oxidative stability [47]. Thus, dietary components may instigate headaches through numerous mechanisms, including influences on neuropeptides, neuroreceptors, and ion channels such as CGRP, as well as inflammatory processes, sympathetic nervous system activation, cortical impacts, nitric oxide release, vasodilation, and shifts in cerebral glucose metabolism [16]. The recent advent of monoclonal antibodies targeting CGRP and its receptors for migraine prophylaxis, alongside CGRP receptor antagonists for acute migraine management, has heralded a new era in migraine therapy [48]. Notably, CGRP appears to be integral to the gut–brain–microbiome axis [44]. Dietary components may influence CGRP secretion and vice versa; CGRP may affect nutrient consumption, appetite, or feelings of fullness by triggering anorexigenic neuropeptides [18]. Thus, CGRP modulates several gastrointestinal tract functions that are susceptible to dietary nutrient regulation. Given the postulated link between migraines and nutrition, there is a compelling case for intensified research focusing on nutrition-centric approaches to migraine management.

This study had several limitations. First, due to its retrospective design, data were sourced from a specific subset of subjects visiting a university medical center that encompasses five hospitals. As a result, the generalizability of our findings to the wider population may be limited, and there could be potential selection bias or unconsidered confounding factors. Second, subjects were selected based on diagnosis codes in the CDW. Comprehensive clinical data detailing headache characteristics were not gathered, given the retrospective nature of the study. To enhance the accuracy of our findings, we targeted patients who had a history of at least two outpatient treatments or at least one hospitalization for each diagnostic code of migraine. Third, subject nutritional data such as dietary intake were not available in our study. Future prospective studies with more expansive sample sizes are essential to elucidate the relationship between migraines and nutritional status, as well as to assess the practicality of using CONUT and PNI scores in clinical settings for migraine patients.

## 5. Conclusions

This study identified a link between nutritional status, as gauged by the objective nutritional indices CONUT and PNI, and migraine susceptibility. These findings demonstrate the potential utility of these scores in predicting migraine risk. Additionally, our results underscore the prospective benefits of nutrition-centric strategies in migraine management. Further prospective investigations are warranted to explore interventions capable of re-versing both objective nutritional indices and migraine symptoms.

## Figures and Tables

**Table 1:** Clinical characteristics of migraineurs versus controls.

	Migraine	Control	ASD
	(*n* = 6603)	(*n* = 90,509)	
Median age, years (IQR)	46 (36, 57)	49 (39, 59)	0.22
Female, *n* (%)	4888 (74.0%)	40,528 (44.8%)	0.59
BMI categories, (*n*, %)			0.03
Underweight (<18.5)	376 (5.7%)	3570 (3.9%)	
Normal (18.5–29.9)	5777 (87.5%)	81,656 (90.2%)	
Obese (≥30)	450 (6.8%)	5283 (5.8%)	
Diabetes mellitus, *n* (%)	535 (8.1%)	7977 (8.8%)	0.03
Hypertension, *n* (%)	1163 (17.6%)	11,443 (12.6%)	0.15
Dyslipidemia, *n* (%)	987 (14.9%)	11,971 (13.2%)	0.05
Angina, *n* (%)	635 (9.6%)	5281 (5.8%)	0.16
AF, *n* (%)	87 (1.3%)	1413 (1.6%)	0.02
Heart disease, *n* (%)	469 (7.1%)	4760 (5.3%)	0.08
Cerebrovascular disease, *n* (%)	1241 (18.8%)	6534 (7.2%)	0.43
Chronic pulmonary disease, *n* (%)	630 (9.5%)	5610 (6.2%)	0.14
Renal failure, *n* (%)	153 (2.3%)	2258 (2.5%)	0.01
Chronic hepatitis, *n* (%)	257 (3.9%)	5353 (5.9%)	0.09
Anxiety disorder, *n* (%)	368 (5.6%)	1311 (1.4%)	0.32
Depression, *n* (%)	953 (14.4%)	2864 (3.2%)	0.59
Sleep disorder, *n* (%)	690 (10.4%)	2420 (2.7%)	0.44
Menopause, *n* (%)	317 (4.8%)	2613 (2.9%)	0.11

ASD, absolute standardized difference; IQR, interquartile range; BMI, body mass index; AF, atrial fibrillation.

**Table 2:** Malnutrition evaluation based on Controlling Nutritional Status (CONUT) and prognostic nutrition index (PNI) scores.

	Migraine	Control	*p*
	(*n* = 6603)	(*n* = 90,509)	
Median CONUT (IQR)	1 (0, 2)	1 (0, 2)	<0.001
CONUT categories, (*n*, %)			<0.001
Absent (0–1)	3750 (56.8%)	65,663 (72.5%)	
Mild (2–4)	2455 (37.2%)	23,009 (25.4%)	
Moderate (5–8)	355 (5.4%)	1502 (1.7%)	
Severe (9–12)	43 (0.7%)	335 (0.4%)	
Median PNI (IQR)	52.1 (47.2, 56.3)	54.7 (51.5, 57.8)	<0.001
PNI categories by original scoring system, *n* (%)			<0.001
Absent (>38)	6356 (96.3%)	89,408 (98.8%)	
Moderate (35–38)	128 (1.9%)	475 (0.5%)	
Severe (<35)	119 (1.8%)	626 (0.7%)	
PNI categories by tertiles, *n* (%)			<0.001
Tertile 1 (<52.55)	3519 (53.3%)	28,844 (31.9%)	
Tertile 2 (52.55–56.59)	1526 (23.1%)	30,844 (34.1%)	
Tertile 3 (>56.59)	1558 (23.6%)	30,821 (34.1%)	

**Table 3:** Multivariate analysis of the association between objective nutritional indices and migraine.

	Unadjusted	*p*	Fully Adjusted	*p*	Backward Elimination	*p*
	OR (95% CI)		OR (95% CI)		OR (95% CI)	
CONUT categories						
Absent (0–1)	1.0 [Reference]		1.0 [Reference]		1.0 [Reference]	
Mild (2–4)	1.87 (1.77–1.97)	<0.001	1.70 (1.61–1.80)	<0.001	1.70 (1.61–1.80)	<0.001
Moderate (5–8)	4.14 (3.67–4.67)	<0.001	4.99 (4.36–5.72)	<0.001	5.00 (4.37–5.74)	<0.001
Severe (9–12)	2.25 (1.63–3.09)	<0.001	3.18 (2.25–4.51)	<0.001	3.21 (2.26–4.54)	<0.001
CONUT per 1-point increase	1.26 (1.25–1.28)	<0.001	1.29 (1.27–1.31)	<0.001	1.29 (1.27–1.31)	<0.001
PNI categories						
By original scoring system						
Absent (>38)	1.0 [Reference]		1.0 [Reference]		1.0 [Reference]	
Moderate (35–38)	3.79 (3.11–4.62)	<0.001	4.76 (3.82–5.94)	<0.001	4.72 (3.79–5.89)	<0.001
Severe (<35)	2.67 (2.19–3.26)	<0.001	3.88 (3.11–4.84)	<0.001	3.87 (3.10–4.82)	<0.001
By tertiles						
Tertile 1 (<52.55)	1.0 [Reference]		1.0 [Reference]		1.0 [Reference]	
Tertile 2 (52.55–56.59)	0.41 (0.38–0.43)	<0.001	0.43 (0.40–0.46)	<0.001	0.43 (0.40–0.46)	<0.001
Tertile 3 (>56.59)	0.41 (0.39–0.44)	<0.001	0.49 (0.46–0.53)	<0.001	0.49 (0.46–0.53)	<0.001
PNI per 1-point increase	0.93 (0.92–0.93)	<0.001	0.92 (0.92–0.93)	<0.001	0.92 (0.92–0.93)	<0.001

Results are expressed as odds ratios (OR) with confidence intervals (CIs).

## Data Availability

Not applicable.

## Appendix A

**Table A1:** Risk of malnutrition based on the CONUT and PNI nutritional scoring systems.

Nutritional Score	Risk of Malnutrition			
	Absent	Mild	Moderate	Severe
CONUT (points)	0–1	2–4	5–8	9–12
Albumin (g/dL)	≥3.5	3.0–3.4	2.5–2.9	<2.5
Score	0	2	4	6
Total cholesterol (mg/dL)	≥180	140–179	100–139	<100
Score	0	1	2	3
Lymphocyte count (×10^9^/L)	≥1.60	1.20–1.59	0.80–1.19	<0.80
Score	0	1	2	3
PNI (points)	>38		35–38	<35
Formula: 5 × lymphocyte count (10^9^/L) + 10× serum albumin concentration (g/dL)				

CONUT, Controlling Nutritional Status; PNI, prognostic nutrition index.
